# A Review of Insulin-Dosing Formulas for Continuous Subcutaneous Insulin Infusion (CSII) for Adults with Type 1 Diabetes

**DOI:** 10.1007/s11892-016-0772-0

**Published:** 2016-07-25

**Authors:** Allen B. King, Akio Kuroda, Munehide Matsuhisa, Todd Hobbs

**Affiliations:** 1 Diabetes Care Center, 1260 S. Main Street, Salinas, CA 93901 USA; 2 Diabetes Therapeutics and Research Center, Institute for Advanced Enzyme Research, Tokushima University, 3-18-15, Kuramoto-cho, Tokushima, 770-8503 Japan; 3 Novo Nordisk, Inc., 100 College Rd W, Princeton, NJ 08540-6604 USA

**Keywords:** Type 1 diabetes, Insulin pump, Dosing formulas, Insulin-dosing guidelines, Continuous subcutaneous insulin infusion

## Abstract

Dosing guidelines for patients with type 1 diabetes using continuous subcutaneous insulin infusion (CSII), which are historically based on clinical experience and retrospective studies of patients consuming an American diet, recommend that basal insulin should represent approximately 50 % of the total daily dose (TDD). Recent prospective studies in the USA and Japan conclude that the more appropriate proportion is closer to 30–40 % of TDD. In addition, currently used formulas for calculating the carbohydrate-to-insulin ratio (CIR) and correction factor (CF) may lead to underdosing of bolus insulin by as much as 12.8–50 % for a hypothetical patient. The discrepancies between traditional formulas and data from newer studies can be accounted for by the more rigorous design of the newer studies (e.g., prospective design, controlled diets, meal omission, and frequent glucose monitoring). International differences in diet composition may also be important to consider when developing dosing recommendations for CSII.

## Introduction

Continuous subcutaneous insulin infusion (CSII) is an intensive therapy typically reserved for motivated patients with type 1 diabetes (T1D) who have frequent hypoglycemia, a significant dawn phenomenon (excess hepatic glucose production and non-hepatic insulin resistance in the morning period) or widely fluctuating blood glucose when using multiple daily injections (MDIs) [1•]. If properly managed, CSII may provide patients with improved glucose control compared with MDI therapy [2, 3] and a lower incidence of severe hypoglycemia [4]. As noted in a recent review, the total number of insulin pump users worldwide is unknown but believed to vary greatly across countries [5]. Estimates suggest that there may be as many as 350,000–515,000 insulin pump users in the USA [1•, 6]. A large registry-based study of the more experienced endocrinology centers in the USA indicated that as many as 50 % of their patients with T1D used a pump [7]. Among European nations, in a 2010 report, the proportion of patients with T1D using CSII varies substantially, from ∼1 % in Russia and Portugal to about 20 % in Norway, Austria, the Netherlands, and Switzerland [8]. A 2011 publication estimated that about 10 % of Australian patients were using CSII, with an increasing number of patients initiating CSII sooner after diagnosis than in previous years [9]. Among Asian nations, the proportion of patients with T1D who are using CSII in Japan is estimated to be 7 % (author communication with Medtronic Japan).

Precise insulin dosing during CSII is necessary to enable patients with diabetes to adhere to current treatment guidelines [10, 11]. Unfortunately, precise dosing is complicated by the need to calculate two to five different basal rates for a 24-h period to match varying insulin needs over the day [12, 13]. Accurate formulas are essential to estimate the initial insulin dose and to adjust insulin pump settings, and these include the total basal dose (TBD), carbohydrate-to-insulin ratio (CIR), and the correction factor (CF; see Table 1). Patients using CSII have insulin requirements that are specific to each individual, and most patients do not have the ability to slowly titrate their insulin up to a safe and effective dose. Over the last decade, rigorously designed studies have been completed that question the accepted methods for insulin-dosing calculation used worldwide. We will discuss this discrepancy and its impact on treating T1D with CSII.

**Table 1:** Definition of parameters used in formulas for establishing proper daily insulin dose in CSII

Parameter	Definition
TDD	The total of basal and bolus insulin dosage in U/day
TBD	The fraction of TDD given as basal insulin in U/day
CIR	A number that, when divided into the number of grams of carbohydrates to be consumed, yields the units of insulin needed to lower blood glucose to a pre-meal level within 2–4 h. CIR is usually between 5 and 20 g of carbohydrates/U
CF	A number that, when divided into the difference between actual and target blood glucose, indicates the number of extra units of insulin necessary to bring glucose within target range, within 2–4 h. CF is usually between 20 and 100 mg/dL/U

*Source*: [14]

Patients with greater insulin sensitivity will have higher CFs and CIRs

*CF* correction factor, *CIR* carbohydrate-to-insulin ratio, *CSII* continuous subcutaneous insulin infusion, *TBD* total basal dose, *TDD* total daily dose

## Where Have Current Dosing Formulas Come From?

The chronological development of published insulin-dosing formulas is presented in Table 2, with a detailed description of their derivation available in a recent review [29•]. Briefly, the first formula proposed in 1922, using a very crude insulin preparation, was weight based and was calculated conservatively in order to avoid hypoglycemia and to preserve the limited amount of insulin available (body weight × insulin sensitivity in dogs [U/kg], divided by 2) [30]. In 1982, Skyler et al., based upon clinical experience using buffered human regular insulin, suggested that the TBD should be about 40 % of the total daily dose (TDD) [15].

**Table 2:** Evolution of dosing formulas for insulin therapy

	Study	TBD	CIR	CF
Clinical	Skyler et al. 1982 [15]	∼40 % TDD		
	Davidson 1982 (see Davidson et al. 2008) [16]			1500/TDD
	Steed 1998 (cited in Davidson et al. 2008) [16]		(3 × wt lb)/TDD	
Retrospective	Davidson et al. 2003 [17]	48 % × TDD	(2.8 × wt lb)/TDD	1724/TDD
	Davidson et al. 2008 [16]	47 % × TDD	441/TDD^a^ or (2.8 × wt lb)/TDD	1694/TDD
	Walsh et al. 2010 [18]	47.6 % × TDD	(2.6 × wt lb)/TDD	1960/TDD
	Alcantara-Aragon et al. 2015 [19•]	58 % × TDD	350–400/TDD	NR
	Pankowska et al. 2008 (pediatric) [20]	27.7 % × TDD	NR	NR
	Alemzadeh et al. 2012 (pediatric) [21]	28 % × TDD	13.5 × BW kg/TDD	2800/TDD
Prospective	King and Armstrong 2007a,b [12, 22]	38.4 % × TDD	(217/TDD) + 3	(1076/TDD) + 12
	Kuroda et al. 2011 [23]	27.7 × TDD	NR	NR
	Kuroda et al. 2012 [24]	27.0 × TDD	300–400/TDD	NR
	King 2010 [25]	40 % × TDD	300/TDD	1500/TDD
	King et al. 2012a [26]	33 % × TDD	90/TBD	NR
	King et al. 2012c [27]	33.9 % × TDD	365/TDD	
	Nakamura et al. 2014 [28•]	27.3 % × TDD	300–500/TDD	

*CF* correction factor, *CIR* carbohydrate-to-insulin ratio, *NR* not reported, *TBD* total basal dose, *TDD* total daily dose

^a^For calculation of TDD, patients with type 2 diabetes were excluded

The next set of formulas, which form the basis for widely used guidelines, were derived from retrospective insulin pump downloads. In 1998, according to Davidson et al. [16], Steed proposed the “rule of 3” for estimating CIR (CIR = [3 × weight in lb]/TDD). Current formulas for deriving CF can be traced to the suggestion by Davidson et al. of the “1500 rule” for calculating CF (CF = 1500/TDD), later revised to “1700” (CF = 1700/TDD) [16]. Together, these three formulas became the foundation of the Accurate Insulin Management (AIM) system. Walsh et al., also using retrospective pump download information, further suggested that CIR be calculated from 450/TDD [18]. Since the previous formulas had been based on regular insulin used in pump treatment, Walsh et al. recommended that they be modified to CF = 2000/TDD and essentially agreed with Davidson that the formula used to estimate CIR should be CIR = (2.6 × weight in lb)/TDD, reflecting the widespread use of rapid-acting insulin analogs [18]. Based on these early studies, current insulin-dosing guidelines in the American Association of Clinical Endocrinologists/American College of Endocrinology (AACE/ACE) consensus statement for the USA [1•] recommend the following formulas: TBD (U) = 50 % of TDD, CIR = 450/TDD, and CF = 1700/TDD.

A few years ago, researchers working independently in Japan and the USA published studies using more rigorous designs and suggested that traditional formulas overestimated basal insulin requirements and underestimated bolus requirements (reviewed in [29•]). Key features of these prospective studies included isocaloric controlled diets, meal omission to study basal rate, timed blood glucose or continuous glucose monitoring (CGM)-guided dose titration, and omission of those individuals with insulin sensitivity that significantly deviated from normal. A strength of the US studies was that CGM allowed for precision in glucose measurement that was unobtainable with intermittent self-monitored blood glucose tests and was able to capture nocturnal and post-meal variations in blood glucose [12, 22]. These data are further supported by a post hoc analysis including patients from two other studies who met the original inclusion criteria [31], resulting in a total of 101 patients [25]. In that analysis, TBD was estimated as 40 % of TDD.

Studies by Kuroda et al. in Osaka and Tokushima, and by Nakamura et al. in Kobe, Japan, suggest that King’s US results may be generalizable to other populations. In the first of these studies by Kuroda et al., TBD was determined to be approximately 30 % of TDD [23]. In their second study, Kuroda et al. established patients’ basal dose and then assessed CIR, finding that CIR exhibited diurnal variability ranging from 300/TDD at breakfast to 400/TDD for lunch or dinner [24]. In another prospective study, Nakamura et al. studied 28 Japanese adults with pump-treated T1D at Kobe University, Japan [28•]. After stabilizing subjects on an isocaloric, fixed diet (50–60 % carbohydrate/20–25 % fat/15–20 % protein), the basal dose was adjusted to 130 ± 30 mg/dL with meal omission and 8-point blood glucose testing. After the basal dose was established, the CIR was established to achieve a postprandial glucose (∼2 h) of <180 mg/dL. Their results indicated that the TBD/TDD should be 27.3 % and the CIR for breakfast should be 300/TDD.

When these prospective studies are combined, the TBD/TDD appears to be much lower than the current recommendation of 50 % and somewhat closer to 30 % (see Table 2). The slightly lower basal dose in the Japanese compared to the US studies may be due to higher-carbohydrate and lower-fat proportions in the Japanese diet. Since most worldwide studies suggest the TDD/kg is around 0.5–0.6 U/kg, the lower basal dose would lead to a higher meal bolus (a lower CIR). Although both Japanese studies recommend a lower CIR in the morning and a higher CIR during the day, all studies confirm that higher bolus doses are necessary.

## Explaining the Discrepancies

Study design issues may help to explain the discrepancy between the results of the King, Kuroda, and Nakamura studies vs. those of earlier work. A major distinction is that these newer results are based on prospective data [12, 13, 22, 23, 25, 28•], whereas the current guidelines are derived from retrospective studies that did not exclude patients who may have experienced transient changes in insulin resistance, for example, due to illness, surgery, or prednisone treatment [16–18]. In the retrospective studies, downloaded pump data were not accompanied by information about patients’ diet, snacks, physical activity, the timing and intensity of their blood glucose monitoring, or post-meal glucose targets, while the prospective studies used controlled diets, self-monitored blood glucose or CGM, and/or frequent clinic visits [12, 13, 22, 23, 25]. The prospective studies also determined patients’ basal rate using sequential meal omissions and their CIR by prescribing meals of known carbohydrate content. CF was determined either by reducing the meal bolus and determining the insulin dose needed to achieve target glucose within 2–4 h, or by clinical judgment.

It should be noted that the CIR and CF values in the studies by Davidson et al. [16, 17] were determined without knowing the proportion of patients who used their bolus calculators and without the ability to confirm whether the pump recommendations were actually followed, and that a linear regression was used for data that may not have met criteria for normality. In Davidson et al., patients’ mean TDD was determined from only 7 days of data [16]. It is important to recognize that patient self-monitoring is a less than ideal way to assess blood glucose over an entire 24-h period; it may not capture nocturnal variations and intermittent, single-point estimates cannot reflect the full variation throughout post-meal periods.

As was the case with the studies by Davidson et al., it is not known whether the pump settings in the studies by Walsh et al. were made based on a standardized diet, whether basal rates were set by studying glucose during meal omissions, whether frequent and timed blood glucose readings were performed, or whether patients with transient changes in insulin sensitivity were excluded. And, although Walsh et al. identified Davidson et al.’s studies as single center, they did not identify their own patient distribution [18].

The ratio of ∼50 % for TBD/TDD used in current formulas is comparable to that reported in clinical trials of basal–bolus therapy in T1D [e.g., 32]. This is not surprising, given that morning fasting plasma glucose is often used as a surrogate for basal insulin needs for the remainder of the day. Titrating to morning fasting glucose may not be the optimum way to determine basal dose, as factors other than inadequate basal insulin (e.g., the “dawn phenomenon,” late-night eating, delayed gastric emptying, and increased hepatic glucose output due to fatty infiltration) may elevate plasma glucose when measured pre-breakfast [14, 28, 33•].

It is also possible that, in the retrospective studies utilizing CSII, post-meal hypoglycemia attributed to excessive bolus dosing may actually have been due to excessive basal dosing. In CSII, basal doses may seem somewhat easier and safer to increase than a bolus dose. In addition, since CGM was not used, nocturnal hypoglycemia arising from excessive basal dosing may not have been recognized. Both of the Kuroda et al. studies used 7–8-point blood glucose measurements rather than CGM and, as a result, may not have captured all post-meal or nocturnal blood glucose variations. As has been noted, it is also unknown whether the single ethnic group in the Kuroda et al. studies and/or the Japanese diet, which contains less fat and more carbohydrates than western diets, may also have affected these results [23, 24, 28•].

## Internationalizing These Findings—Should Dietary Patterns Matter?

The dosing guidelines in CSII in India [34] and China [35] recommend the same dosing formulas as AACE/ACE, i.e., TBD = 50 % of TDD and CIR = 450/TDD. This seems questionable because the diet composition in these nations is markedly different from the US diet that was probably consumed in the Davidson and Walsh studies. The American diet is approximately 49 % carbohydrate and 38 % fat, compared to 61 % and 27 %, respectively, in China, and 71 % and 19 %, respectively, in India [36].

How might dietary carbohydrate differences affect dosing formulas across different cultures? The denominator for the TBD formula, TDD, is the summation of the TBD and total bolus dose. Increasing the dietary carbohydrates would increase the total bolus dose, increasing the TDD but not the TBD, resulting in lower recommendations for the TBD/TDD ratio. For example, every 10 % increase in dietary carbohydrates and assuming 1800-calorie diet, CIR = 10 g/U, no corrective boluses, and a TBD = 10 U, there would be a 10 % decrease in the TBD/TDD ratio.

Given that a high-fat, low-carbohydrate intake reduces the ability of basal insulin to suppress hepatic glucose production [37], it could be assumed that the US recommendations for basal dose would be higher than in countries with lower-fat content in their diet. For the Asian Indian diet, for example, consisting of 71 % carbohydrates and 19 % fat, the total bolus dose should be much higher than the US diet, but the total basal dose would be less. When considering the combined effects of increased dietary carbohydrates, reduced fat, and the possible excessively high recommendations based on retrospective studies, the recommendation of TBD to be 50 % of TDD seems excessive for India, China, and other countries of similar diet composition. The TBD/TDD ratio of <30 % in the Japanese studies supports this contention [23, 24, 28•].

## How Have Existing Guidelines Embraced This New Information?

Very little new empirical research focusing on insulin dosing in CSII in T1D has been published since the King and Kuroda studies described earlier [i.e., 12, 22–25]. Nevertheless, one retrospective study of 170 pump-treated patients with T1D from Spain that focused on establishing the CIR found that the mean CIR in the morning was 350/TDD for breakfast and 400/TDD for other meals [19•]. The TBD/TDD reported in that study was quite high, 58 %, and it was not stated whether the TBD had been set by standardized diet, intense glucose monitoring, and meal omissions.

There have been several papers about insulin dosing in type 2 diabetes (T2D) [38–41]. In China, in a study with 65 newly diagnosed patients with T2D treated with an insulin pump, TBD was about 40 % of TDD [40], whereas, in another study comparing MDI and CSII (100 patients in each group), TBD was 50.9 % in the MDI group vs. 69.1 % in the CSII group [40]. Interestingly, the actual TBD/TDD ratio in patients using MDI basal–bolus therapy was about 20 % in another uncontrolled study [38]. C-peptide represents the residual insulin secretion, and it is not usually depleted in patients with type 2 diabetes. Pankowska et al. compared the basal insulin requirement in C-peptide-positive and C-peptide-negative patients with type 1 diabetes [20]. TBD/TDD ratio was 0.32 in C-peptide-negative patients and 0.23 in C-peptide-positive patients. Insulin secretion might reduce the basal insulin requirement. So, the TBD/TDD in patients with type 2 diabetes might be lower than TBD/TDD in patients with type 1 diabetes. More vigorous studies [e.g., 27] should answer this question.

## What Should Be Done in High-Quality Studies to Further Encourage Adoption of These Revised Formulas—What Else Is Needed?

Accurate formulas are essential to estimate the initial insulin dose and to adjust insulin pump settings. For formulas to be accepted, we believe that they should be based on studies with the following attributes:

1. An adequate number of representative patients.
2. Excluding patients who have altered insulin sensitivity, such as those with renal failure or on corticoid therapy.
3. Diet of known composition that reflects that of the country in which the recommendations are targeted. The fat (increased insulin resistance and delayed gastric emptying) and protein content (gluconeogenesis and increase glucagon) have a significant effect on the estimation of dosing factors [33•, 42, 43, 44•].
4. Meals omitted at various times to isolate and adjust the basal insulin dose first.
5. After the basal dose has reached goal, determine the CIR by the amount of insulin necessary to return the post-meal glucose to target following a meal of known carbohydrate content.
6. The plasma glucose must be monitored by focused timed and frequent self-monitored blood glucose or, ideally, CGM.
7. The expected variability of a formula result should be stated to allow the provider to judge the appropriateness of a calculated dosing parameter.

## Moving Forward

Data from several modestly sized but carefully controlled prospective trials in Japanese and American populations suggest that current widely used insulin-dosing recommendations should be reconsidered, because existing formulas are likely to result in an excess basal insulin dose and an insufficient bolus dose. We recommend a revised set of formulas to estimate dosing for insulin pump-treated patients with T1D. For example, for a hypothetical patient with a TDD of 50 U consuming a 70-g carbohydrate meal, under existing formulas (TBD (U) = 50 % of TDD, CIR = 450/TDD, CF = 1700/TDD [1•]), the bolus dose would be ∼7.8 U and the TBD would be ∼25 U. Using the revised formula (TBD = 30–40 % TDD and CIR = 300–400/TDD) results in a bolus dose of 8.8–11.7 U and a TBD of 15–20 U. This difference amounts to anywhere from a 12.8–50 % increase in prandial bolus dose and a 20–40 % decrease in TBD.

It remains to be seen whether the estimated range for the CIR (i.e., 300–400) can be further narrowed and whether the diurnal variability of CIR should be taken into account. It has been reported that higher insulin boluses are needed for the first meal of the day [24, 28•, 45], while others still have not found this to be the case. King et al. found that suppression of the earlier-than-breakfast hepatic glucose production mitigated the increased need for higher insulin doses during that time period [12, 22]. Hinshaw et al. determined, through extensive testing, that, while there may be an increase in insulin resistance in the morning, the marked within-subject variability obliterated any statistical difference [46]. More studies using intensive glucose monitoring (such as CGM) for full suppression of the dawn phenomenon are needed to determine whether there is any significant change in bolus-dosing factors associated with the meals of the day.

There has only been one reported study of the direct assessment of CF. King and Armstrong [22] gave only 75 % of the predicted bolus for a meal. After 4 h post-meal, the resulting hyperglycemia was treated by a bolus of insulin adjusted to return the interstitial glucose to within 20 % of a target of 100 mg/dL within 4 h. They found that, when the slope of the relationship between CF and the 1/TDD is forced through zero, the CF-estimating formula would be 1407/TDD (*R*^2^ = 0.620). The ratio CF/CIR was 4.44 (*R*^2^ = 0.900). This tight relationship exceeded that of deriving the CF from 1/TDD, suggesting that, if CIR is correctly determined, 4.44 × CIR would be a better estimate then the rounded relationship of 1400/TDD. A greater bolus to treat episodic hyperglycemia is in keeping with the greater bolus for meal carbohydrates. However, more studies are required to support these findings.

We have also shown that the revised formulas can inform insulin dosing in patients with T1D using MDI therapy [13, 26]. It is important to note that with once-nightly injection of a basal insulin analog, one cannot obtain a flat baseline glucose level during the dawn phenomenon period without increasing the risk for hypoglycemia during the non-dawn periods [13, 26, 47]. On the other hand, in pump-treated T2D when vigorously studied with diet control and CGM titration, one basal rate was successful in achieving nearly ideal basal control, 99 mg/dL, in 80 % of patients [27]. The TBD was only 0.22 U/kg. With respect to once-daily basal insulin injection regimens in T2D, in 11 treat-to-target clinical trials with neutral protamine Hagedorn (NPH) insulin or insulin glargine, maintaining a mean morning fasting glucose of <110 mg/dL required an average basal insulin dose of >0.6 U/kg [48•]—a dose nearly three times greater than that suggested using our revised formula (∼0.2 U/kg). Again, such disparities have enormous clinical implications for patients. As the revised formulas are based on research in T1D patients only, broad applicability for T2D patients is unknown. However, there may be some utility in guiding clinicians when initiating or intensifying basal–bolus insulin therapy in these patients as well, paying close attention to individual patient factors such as residual beta-cell function and degree of insulin resistance. Clearly, comparative studies of different formulas are needed in these patients as well.

## Conclusions

Further studies are warranted to explore insulin dosing in patients with diabetes, during which meals are controlled and glucose intensely monitored. Ideally, these should be prospective and designed to allow head-to-head comparison of different dosing formulas. This would also provide important information about differences in hypoglycemia or hyperglycemia with different dosing formulas. Finally, regardless of which formulas are used, it is important to emphasize that recommended insulin-dosing formulas are intended as estimates, and should always be tempered with clinical judgment, based upon experience, according to patient-specific factors including diurnal variation in blood glucose and activity level. For example, it is possible that some patients might require more than one CIR over a 24-h period to achieve optimal glycemic control and prevent nocturnal hypoglycemia. Until appropriate studies are published, we believe that new evidence indicates that the following revised formula should be considered as a starting point when initiating a patient with T1D on intensive therapy: TBD = 30–40 % of TDD and CIR = 300–400/TDD.
